# Prevention of Preterm Birth with Progesterone

**DOI:** 10.3390/jcm10194511

**Published:** 2021-09-29

**Authors:** Gian Carlo Di Renzo, Valentina Tosto, Valentina Tsibizova, Eduardo Fonseca

**Affiliations:** 1 Centre of Perinatal and Reproductive Medicine, Department of Obstetrics and Gynecology, University of Perugia, 06132 Perugia, Italy; tosto.valentina@libero.it; 2 Department of Obstetrics and Gynecology, Faculty of General Medicine, I.M. Sechenov First State University of Moscow, 119991 Moscow, Russia; 3 Almazov National Medical Research Centre, Health Ministry of Russian Federation, 197341 Saint Petersburg, Russia; tsibizova.v@gmail.com; 4 Department of Obstetrics and Gynecology, Federal University of Paraiba, Joao Pessoa 58051-900, PB, Brazil; fonseca2003@yahoo.com

**Keywords:** preterm birth, risk factors, prevention, 17-OHPC, micronized progesterone, perinatal outcomes, recommendations

## Abstract

Gestational age at birth is a critical factor for perinatal and adulthood outcomes, and even for transgenerational conditions’ effects. Preterm birth (PTB) (prematurity) is still the main determinant for infant mortality and morbidity leading cause of infant morbidity and mortality. Unfortunately, preterm birth (PTB) is a relevant public health issue worldwide and the global PTB rate is around 11%. The premature activation of labor is underlined by complex mechanisms, with a multifactorial origin influenced by numerous known and probably unknown triggers. The possible mechanisms involved in a too early labor activation have been partially explained, and involve chemokines, receptors, and imbalanced inflammatory paths. Strategies for the early detection and prevention of this obstetric condition were proposed in clinical settings with interesting results. Progesterone has been demonstrated to have a key role in PTB prevention, showing several positive effects, such as lower prostaglandin synthesis, the inhibition of cervical stromal degradation, modulating the inflammatory response, reducing gap junction formation, and decreasing myometrial activation. The available scientific knowledge, data and recommendations address multiple current areas of debate regarding the use of progesterone in multifetal gestation, including different formulations, doses and routes of administration and its safety profile in pregnancy.

## 1. Introduction

Preterm birth is defined by the World Health Organization (WHO) as delivery before 37 completed weeks of gestation. About 85% of these premature births occur at 32 to 36 weeks, 10% are born at 28–31 weeks, and 5% at <28 weeks of gestation (extremely preterm babies).

Being born too soon is an important cause of infant deaths from prematurity every year, and many preterm newborns have long term disabilities. About 15 million babies are born preterm annually, with an increasing trend in cases rates worldwide, putting the global PTB rate at 11% [1,2,3].

There is growing evidence that the progesterone can be useful in high risk pregnancies for preterm birth. The use of progestogens has been extensively studied over the years and it is still a topic of interest in current research [4,5,6,7,8,9,10,11,12,13]. Expert researchers suggest that the rate of PTB may be reduced by the prophylactic use of progesterone, especially in women with a high risk profile, including a previous history of spontaneous preterm delivery and in women revealed to have a short cervical length (CL) at transvaginal ultrasound (TVU) [14].

The complex pathogenesis of preterm labor activation makes reliable prediction difficult [15]. An obstetric history of spontaneous preterm birth (sPTB) is considered the strongest predicting factor. sPTB recurs in 35 to 50% of pregnancies, and the risk of a recurrent event recurrence is proportional to the number of prior spontaneous preterm deliveries. Several other risk factors have been associated with at the evidence surrounding the variability of this obstetric event, including non-Hispanic Black race, low socioeconomic status, midtrimester cervical length <25 mm, cervical-vaginal infections, history of cervical surgery procedures, maternal smoking, poor or no prenatal care, uterine overdistension, decidual hemorrhage, and short interpregnancy interval. Others conditions possibly associated with spontaneous preterm birth are multiple pregnancy, pregnancy derived by assisted reproductive techniques (ART), periodontal disease, maternal anemia, environmental factors and epigenetics [16]. Recently, experts advocated that a possible association may exist between environment and preterm birth. How pollution and other contaminants may induce maternal-fetal effects is still unexplained; however, some researchers have demonstrated the probable influence of air pollution on epigenetic effects. Other recent scientific evidence suggests that epigenetics may, in turn, be linked to preterm labor [17].

Table 1 lists the known risk factors, the level of association and the possible interventions for PTB.

Extensive data reported that the ultrasound measurement of CL at mid-gestation may be a useful strategy for predicting the risk of pretem birth delivery for both singleton and twin pregnancies [18,19,20,21,22]. A growing body of studies agree that the administration of progestogens to high-risk women, mainly with a singleton pregnancy, significantly reduces the rate of sPTB [4,5,6,7,8,23,24,25]. A lack of strong evidence and controversial opinions exist on progesterone’s usefulness in twin/multifetal pregnancies.

## 2. Progesterone: Biochemical “Identikit” and Rationale for Use

The history of progesterone (PG) is long and characterized by numerous and fascinating chronological steps, and it is probably destinated to have a “never-ending history”, as discussed in a recent paper [26,27]. Progesterone is probably the oldest hormone scientists know about. The terms “progestogens” or “progestagens” refer to natural or synthetic chemical forms with progestational activity.

Nowadays, the pharmacokinetic and pharmacodynamic features of progesterone are well-known.

The understanding of the pharmacodynamics of progesterone in preterm labor prevention is based on the evidence that it relaxes the uterus throughout pregnancy by inhibiting the expression of estrogen receptor alpha (ER-a) and reducing sensitivity to estrogen [28]. Overall, progesterone shows numerous functions on the myometrium: it has been shown to induce high levels of cyclic adenosine mono phosphate (cAMP) and time-dependent stimulation of nitric oxide synthetase (NOS), as well as to inhibit the myometrial gap junctions’ (channels made of connexin 43) formation. Natural progesterone (P4) and its metabolites promote uterine quiescence both through interactions between nuclear and membrane P4 receptors and by inducing low levels of the inflammatory prostaglandins (via cyclooxygenase), oxytocin and intracellular calcium [29,30,31,32].

The route of administration seems crucial in determining the optimal pharmacodynamic profile of P4 and in obtaining the desired clinical effects. Most of the body of scientific evidence regards the vaginal, intramuscular and oral routes, as discussed below. The most important revolution in theme of progesterone is the development of micronization process, which led to further optimization of clinical effects and objectives deriving from its use. The micronization of progesterone and its suspension in oil-filled capsules was first studied in the late 1970s; this allowed progesterone to be absorbed more efficiently by the traditional oral route [26].

Nowadays, micronized progesterone products are largely preferred and used in obstetrics (and not only in this field) for many medical conditions, including threatened miscarriage, recurrent pregnancy loss and PTB prevention [26,33,34]. Based on the major part of consistent researches, the vaginal administration would be the best option to use due to the better concentrations that reach the uterus for the “first uterine pass effect” and to avoid the unwanted side effects such as nausea, headache, and sleepiness derived from oral route [26,35]. Table 2 reports the main e biochemical, immune and hormonal mechanisms of progesterone that are involved in the maintenance of pregnancy.

## 3. Progesterone and PTB: Where We Are Now

The current best approach to limit the preterm birth burden is based on early detection risk and prevention by multilevel and even combined strategies. In recent years, international and national societies have shown a great need to identify and manage women at risk of delivering prematurely, developing and sharing recommendations that are not absolute but which are, nevertheless, useful [36].

### 3.1. Identification of PTB High Risk Women

The traditional method of antenatal screening is based on an accurate history based on factors such as maternal age, race, smoking status, and previous and current obstetric history. The available risk scoring systems, which attempt to define the pregnancy’s level of risk, have been shown to have a scarce detection rate and a high false-positive rate [37]. An alternative strategy is to identify high risk women by cervical length measurement at 20–24 weeks of gestation [14,18,19]. Studies revealed that in both singleton [14,18,19] and twin pregnancies [14] the rate of early spontaneous birth can be predicted from the measurement of CL in this gestational period.

Thus, measuring cervical length by TVU is a simple and effective test for the prevention of PTB, but routine CL screening is not clearly recommended by some international societies [37,38,39,40,41]. Furthermore, both the American College of Obstetricians and Gynecologists (ACOG) and the Society for Maternal–Fetal Medicine (SMFM) recognize that such a screening strategy may be considered [38,39,40].

The FIGO Working Group on Best Practice in maternal-fetal medicine was clear in its statement in favor of the universal screening of pregnant women by mid-trimester TVU evaluation of the cervical length as a useful intervention to decrease preterm births in pregnant women with a short cervix [36,42]. Similarly, the European Association of Perinatal Medicine had approved the universal CL screening as a relevant PTB detection strategy [43].

Once risk assessment is established, the choice on how to manage risk may involve one or a combination of several preventive strategies. Regarding progesterone use, differences exist worldwide.

### 3.2. Management of Short Cervix in Singleton Gestation with NO History of sPTB

Given the association of cervical shortening with preterm birth, several interventions aimed at decreasing the PTB rate have been investigated, including intramuscular 17-hydroxyprogesterone caproate (17-OHPC), cerclage, cervical pessary, and vaginal progesterone [9,44,45,46,47]. Studies of pessary and cerclage have produced conflicting results, while 17-OHCP treatment has failed to demonstrate benefit when prescribed for the indication of cervical shortening. The results have been consistently more beneficial and salutary for vaginal progesterone route. In a large multicenter trial, women with a cervical length <30 mm at 16 to 22 weeks were randomized to receive weekly injections of 17-OHPC or placebo. The rate of preterm delivery was similar between groups (25.1% vs. 24.2%, RR 1.03, 95% CI 0.79–1.35), and no improvement was seen in neonatal outcomes [45]. Two smaller studies produced conflicting results on the efficacy of 17-OHPC in the setting of short cervix, with one study demonstrating benefit similar to vaginal progesterone and the other demonstrating no advantage in preterm birth rate reduction. The efficacy of vaginal administration in women with a sonographic diagnosis of short cervix has been reported by two multicenter, randomized controlled trials and by independent patient-level meta-analyses that included data from these studies and several smaller trials. Fonseca et al. conducted a double-blind trial that randomized women with a cervical length ≤15 mm to 200 mg vaginal progesterone or placebo [5]. A total of 413 women were treated from 24 to 34 weeks’ gestation. Delivery prior to 34 weeks was reduced to 19.2% in the group that received vaginal progesterone vs. 34.4% in the placebo group (RR 0.56, 95% CI 0.36–0.86). Eighty-five percent of the women included in this study had no history of preterm birth. In a subgroup analysis of these women, a relevant reduction in preterm birth rate at <34 weeks was noted in women with a short cervix (≤15 mm) who received progesterone (RR 0.57, 95% CI 0.35–0.93) [5]. The PREGNANT trial reported that administration of vaginal progesterone gel (dose of 90 mg) in a pregnant group with a cervical length of 10 to 20 mm identified at mid-gestation resulted in a significant reduction in PTB rate at <33 weeks of gestation (8.9% vs. 16.1%, RR 0.55, 95% CI 0.33–0.92) [48]. Moreover, this study showed neonatal benefits, with a significant reduction in respiratory distress syndrome (RR 0.39, 95% CI 0.17–0.92). Only 16% of the enrolled population had a history of previous PTB, and even after excluding these subjects, progesterone remained associated with a relevant benefit in the setting of isolated short cervix (RR 0.50, 95% CI 0.27–0.90) [48]. A 2018 meta-analysis incorporated data on 974 singleton pregnancies with a CL ≤25 mm and described a decreased risk of preterm birth at <32 weeks of gestation (RR 0.64, 95% CI 0.48–0.86) with vaginal progesterone treatment; preterm deliveries at <28, <34, and <37 weeks of gestation were reduced as well. In addition, the meta-analysis showed a reduction in neonatal morbidity and mortality (RR 0.59, 95% CI 0.38–0.91), as well as a reduction in birthweight <2500 g and <1500 g [46]. Treatment with vaginal progesterone following the diagnosis of a short cervix and the threshold of cervical length at which to start treatment remain areas of debate. The US Food and Drug Administration (FDA) did not approve vaginal progesterone for the indication of preterm birth prevention in the setting of short CL, in part because data from the PREGNANT trial failed to demonstrate a benefit when only US patients were analyzed. In addition, the FDA declined approval because vaginal progesterone did not appear to be effective in Black or obese women. Despite debate about the clinical utility in all subgroups, given the data on the potential benefit and lack of harm, the ACOG and SMFM have recommended vaginal progesterone as a useful strategy for pregnant women with a short cervix [39]. In addition, with evidence of the benefits of vaginal progesterone administration in the setting of a short cervix and its cost-effectiveness, some experts have recommended universal cervical length screening for asymptomatic women without a prior preterm delivery [48,49,50]. The cost-effectiveness of such recommendations, however, is founded on a single, not serial, cervical length measurement at the time of mid-trimester ultrasound examination [51].

### 3.3. Management of Short Cervix in Singleton Gestation with History of sPTB

The National Institute of Child Health and Human Development (NICHD) Maternal–Fetal Medicine Units Network conducted a multicenter double-blind randomized controlled trial of 463 women with a singleton pregnancy and prior spontaneous preterm birth between 16 and 36 weeks who received 17-OHPC or placebo. Treatment with 17-OHPC was associated with a 34% reduction in recurrent preterm birth at <37 weeks of gestation (from 54.9 to 36.3%), as well as significant reductions at <32 and <35 weeks and decreased infant complications, such as intraventricular hemorrhage, necrotizing enterocolitis, and need for supplemental oxygen [13]. In 2011, the FDA approved 17-OHPC for prevention of recurrent preterm birth and it became the standard of care in the United States. More recently, research showed that 17-OHPC administration did not reduce the rate of preterm birth at <37 weeks of gestation (17-OHPC 11% vs. placebo 11.5%), nor did it reduce neonatal morbidity (5.6% vs. 5.0%) [52]. This study could not recruit well in the USA because 17-OHPC was already on the market and incorporated into the standard care for women with prior PTB, resulting in significant demographic and risk differences between the PROLONG trial and the NICHD study. Only 22% of patients enrolled in PROLONG were from the USA; 61% were from Russia and Ukraine. In addition, only 1.1% of patients had a cervical length <25 mm and only 7% of the patients were Black—two of the greatest risk factors for preterm birth. These differences in study populations and the conflicting results of the two trials have introduced considerable uncertainty and controversy into the management of patients with prior preterm birth with 17-OHPC. The FDA convened an advisory panel to review the data on 17-OHPC. The panel voted to recommend that the drug be removed from the market [53]. In October 2020, the FDA Center for Drug Evaluation and Research proposed withdrawal of 17-OHPC from the market. The final decision is subject to potential additional public hearings and a ruling from the FDA Commissioner [54].

A 2020 statement from SMFM concludes that providers can reasonably continue to use 17-OHPC in women with a risk profile similar to that of the enrollees in the NICHD study [55]. Given the preponderance of data in a USA population, the SMFM states that women with a singleton gestation and a history of prior sPTB may be prescribed intramuscular administration of 250 mg 17-OHPC weekly, starting at 16 to 20 weeks of gestation until 36 weeks of gestation or delivery. If 17-OHPC is not available or the patient declines this option, vaginal progesterone may be a reasonable alternative [55].

The clinical advantage of vaginal progesterone has been largely investigated and supported in women with prior spontaneous preterm delivery. While subsequent studies have produced mixed results, a 2019 meta-analysis readdressed the question of the optimal intervention for women with a prior preterm birth and confirmed that vaginal progesterone treatment was associated with a reduction in recurrent preterm birth at <34 weeks (OR 0.29, 95% CI 0.12–0.68) and <37 weeks (OR 0.43, 95% CI 0.23–0.74) [56].

At the present, the debate on intramuscular 17-OHPC, vaginal progesterone and which route and formulation is better is still open. The EPPPIC study group reported results of a meta-analysis, in which data from 31 trials were included. The authors considered trials of both singleton and multifetal pregnancies comparing vaginal, intramuscular and oral progesterone administration with control, or with each other. Compared with controls, both the vaginal route and the intramuscular 17-OHPC reduced the risk of PTB before 34 weeks for singleton pregnancies in high risk women, with a 22% reduction in the relative risk (RR) for participants who received vaginal progesterone (nine trials, 3769 women), and 17% reduction for those received 17-OHPC (five trials, 3053 women) [57]. Importantly, given that the upper confidence limit crosses the line of no effect, the reported implication that 17-hydroxyprogesterone caproate (17-OHPC) “reduced birth before 34 week in high-risk singleton pregnancies” is not justified in light of its lack of statistical significance. This erroneous conclusion could have serious consequences, as 17-OHCP does not have a good safety profile. It is well-established, including randomized evidence, that 17-OHCP causes gestational diabetes mellitus [58], a condition with adverse maternal and neonatal outcome. 17-OHCP is also associated with higher group B streptococcus (GBS) maternal colonization (a well-known contributor to neonatal morbidity and mortality) compared to vaginal progesterone administration [59]. There is also a known increase of cancer in the offspring of mothers treated with 17-OHCP, as reported at the Endocrine Society’s recent annual conference [60].

Regarding the utility of oral progesterone administration as preventive strategy in high risk patients, evidence to support its use in clinical routine practice is still inconsistent [57].

A recent study reported that oral progesterone appears to be effective for the prevention of recurrent preterm delivery and reduction in perinatal morbidity and mortality in asymptomatic singleton pregnancies compared with placebo. More adverse effects with oral progesterone therapy compared with placebo were reported, although none were serious. Thus, future randomized studies comparing oral progesterone with other available therapies for the prevention of recurrent preterm birth are needed [61].

### 3.4. Multiple Pregnancy

Twin pregnancies are associated with a several-fold greater perinatal mortality than singleton pregnancies. PTB Prematurity is an important contributor, with about 50% of twin pregnancies delivering before 37 weeks and 10% delivering before 32 weeks [62]. Trials in unselected twin pregnancies reported that use of progesterone from mid gestation had no relevant effect on reducing prematurity for twins. Just recently, a multicenter trial conducted at 22 European hospitals was published. Women with twin pregnancy were randomly assigned to receive either progesterone (early administration of 600 mcg/daily vaginal progesterone from 11 to 14 weeks) or placebo, and in the random-sequence generation, there was stratification according to the participating center. The results reveal that universal treatment with vaginal progesterone does not reduce the incidence of spontaneous birth between 24^+0^ and 33^+6^ weeks’ gestation. Post hoc time-to-event analysis led to the suggestion that progesterone may reduce the risk of spontaneous birth before 32 weeks’ gestation in women with a cervical length of <30 mm, and it may increase the risk for those with a cervical length of ≥30 mm [63]. In conclusion, there is no strong evidence of the benefit of using universal vaginal progesterone to decrease prematurity in multiple pregnancies. One meta-analysis showed a benefit in reducing adverse perinatal outcomes in a subgroup of women with a short cervix ≤25 mm, suggesting it may be useful in this group, but the study design had several limits and further research is needed. The NICE guidelines for multiple pregnancy followed by UK healthcare providers do not promote the routine use of cervical cerclage or progesterone for the prevention of PTB in multiple pregnancies.

### 3.5. Combination of Preventive and Therapeutic Strategies

Data regarding the efficacy of combining different approaches, such as intramuscular progesterone with or without vaginal progesterone and with or without cervical cerclage and Arabin pessary, are limited, not unanimous, but in evolution. The utility of combined treatment with history-based cerclage and intramuscular 17-OHPC is unclear, and small retrospective studies have reported mixed results [64,65]. The SMFM recommends continuation of 17-OHPC in women who receive an ultrasound-indicated cerclage [66]. Most recently, Shor et al. said that a combined rescue therapy including vaginal progesterone, cervical cerclage, and Arabin cervical pessary emerges as a promising management strategy in pregnant women who have a short cervical length and a high background risk for preterm delivery [67]. The possible advantages of a combined approach were also considered for twin pregnancies. In this regard, a recent study evaluated the efficacy of a combined approach (vaginal progesterone plus cervical pessary) and of vaginal progesterone only in twin pregnancies: the combined use of Arabin cervical pessary and vaginal progesterone in twin pregnancy with short CL may have a synergic and beneficial effect in preventing preterm labor [68]. On the opposite, D’Antonio et al. observed that cervical pessary, progesterone and cerclage do not show a significant effect in reducing the rate of PTB or perinatal morbidity in twins, either when these strategies are applied to an unselected population of twins or in pregnancies with a short cervix [69]. Further research is needed to confirm whether or not these preliminary data both in singleton and twin pregnancies.

## 4. Progesterone Safety Profile

The safety profile of progesterone on newborn health is another topic of growing interest among experts. Many questions remain unexplained, particularly the long-term safety concerns and also whether the use of progestagens may or not improve neonatal and childhood outcomes.

The results of a meta-analysis suggest that the administration of progestogen for preterm birth prevention does not appear to negatively affect neonatal mortality in single or multiple pregnancies regardless of the route of administration [70]. Moreover, similar conclusions were derived from a study on twins, in which the authors found that antenatal exposure to progesterone given in twin pregnancies has no significant impact on child health and developmental outcomes at three to six years [71].

A recent systematic review examined the potential long-term effects of prenatal progesterone treatment on child development, behavior and health: the authors did not find evidence of benefit or harm in offspring prenatally exposed to progesterone treatment for PTB prevention [72]. There is a need for future follow-up studies on prenatal progesterone administration and its effects in offspring beyond early childhood. Therefore, nowadays, the safety profile of natural progesterone is quite confirmed, while the one of progestogens is raising many issues of concern (particularly relating to the use of 17-OHPC and dydrogesterone as alternatives to natural progesterone) [32,35,60].

## 5. Conclusions

The current scientific and clinical evidence suggest that cervical length screening and vaginal progesterone use, eventually combined with cervical cerclage or Arabin pessary, may help to contain or reduce the burden of preterm delivery birth, when used in the appropriate target populations of pregnant women. The current data and recommendations address multiple controversial topic areas regarding the role of progesterone for PTB prevention in multi-gestational pregnancies multifetal gestation, its different formulations, dosages, routes of administration and safety profile in pregnancy. Overall, a growing body of studies are in agreement in identifying progesterone, especially the vaginal formulation, as a keystone among the preventive PTB strategies in well-defined high-risk categories.

## Figures and Tables

**Table 1:** Risk factors, level of association with spontaneous PTB, available preventive-therapeutic interventions [15,16,17,18,19,20,21,22].

Risk Factors	Associations with Spontaneous PTB	Available Interventions
Ethnicity (black)	X	No
Maternal age (young age and advanced age)	X	Yes
Domestic violence	XX	Yes
Low socioeconomic status	XX	?
Stress, despression, negative life events	XX	Yes
Hard work	XX	Yes
No or poor prenatal care	XX	Yes
Smoking, substance abuse (cocaine)	X	Yes
Alcohol, caffeine	X	Yes
Pre-pregnancy BMI, weight gain in pregnancy	X	Yes
Previous preterm delivery or second trimester pregnancy loss	XXX	Yes
Previous cone biospy/cervix surgery	XX	?
Previous cesarean section	X	Yes
Mullerian abnormalities	X	No, Yes or ?
Parity (nulliparity?)	X	-
Short inter-pregnancy interval (<12 months)	X	Yes or ?
Family history for PTB, genetics	X	No
Male baby	X	No
Reproductive system disorders, treatments, ART	X	Yes
Maternal medical disorders (preeclampsia, diabetes, others)	X	No, Yes or ?
Multiple pregnancy	XXX	Yes
Vaginal bleeding	X	No or?
Cervico-vaginal infections	XX	Yes
Uterine contractility	X	Yes
Short cervix/Cervical modification (during antenatal surveillance)	XX	Yes
Periodontitis	X	Yes
Maternal anemia	X	Yes
Environmental factors and epigenetics	X	?

-: not applicable; PTB: preterm birth; ART: assisted reproductive technologies; BMI: body mass index; X: weak demonstrated association; XX: mild demonstrated association; XXX: strong demonstrated assoccation; ?: as-yet unidentified interventions.

**Table 2:** Pharmacodynamic identikit of progesterone in pregnancy maintenance [26,27,28,29,30,31,32].

Biochemical, Immune and Hormonal Effects	+	−
Maternal immune responses modulation (fetus as semiallogenic transplant—needs protection)	+	
Utero-placental perfusion changes and improvements	+	
Myometrial/uterine relaxation through:	+	
-Estrogen receptors (ER-alpha) expression		−
-Estrogen sensitivity		−
-Oxytocin receptors antagonization		−
-Levels of cyclic adenosine mono phosphate (cAMP)	+	
-Nitric oxide synthetase (NOS)	+	
-Formation of myometrial gap junctions (channels made of connexin 43)		−
Cervix integrity promotion	+	
Suppression of fetal immunoplacental inflammatory response	+	
Cervix ripening	+	
CRH (corticotrophin releasing hormone) and cortisol levels		−
Prostaglandins release		−
Vaginal microbiota influence (for vaginal administration)	+	

## Data Availability

The data presented in this paper are available in public datasets at doi, reference number.
